# Missed Bilateral Atraumatic Sacral Ala Insufficiency Fractures With Normal Dual-Energy X-ray Absorptiometry (DEXA) Scan and Radiofrequency Echographic Multi-spectrometry (REMS)-Confirmed Osteoporosis

**DOI:** 10.7759/cureus.101915

**Published:** 2026-01-20

**Authors:** Armaghan Shahid, Banah Khoshnaw, Ibrahim Rakha, Sedeek Mosaid, Paul Lee

**Affiliations:** 1 Trauma and Orthopaedics, Lincoln County Hospital, Lincoln, GBR; 2 Trauma and Orthopaedics, Chelsea and Westminster NHS Trust, London, GBR; 3 Internal Medicine, NHS Lothian, Edinburgh, GBR

**Keywords:** bmd, dexa, diagnostic challenges, fragility fractures, osteoporosis, radiofrequency echographic multi-spectrometry, rems, sacral insufficiency fractures

## Abstract

Sacral insufficiency fractures (SIFs) are often missed in elderly patients, resulting in increased morbidity and mortality. Osteoporosis diagnosis can minimise missing SIFs and other fragility fractures. Dual-energy X-ray absorptiometry (DEXA) remains the gold standard for osteoporosis diagnosis; however, it has its limitations, including reduced accuracy in patients with degenerative spinal changes and prosthetic implants. This case report explores how we can utilise radiofrequency echographic multi-spectrometry (REMS) as an alternative diagnostic tool for osteoporosis.

A 75-year-old woman with a history of bilateral (B/L) total hip replacements and chronic back pain presented with increasing low back and hip pain. Initial imaging included pelvic X-rays, which did not show any fractures, and the patient was discharged with analgesics. Patient symptoms persisted, leading to an MRI, which showed B/L sacral ala insufficiency fractures. Subsequently, a DEXA scan was done, which showed a T-score of -0.9, indicating normal bone mineral density (BMD). Given the suspicion of osteoporosis clinically, REMS was done, which showed significant fragility of the bones, a fragility score of 76/100, hence confirming osteoporosis.

The patient was initially discharged with oral painkillers and free to mobilise as tolerated. After an MRI confirmed B/L SIF, conservative treatment with analgesics and bed rest with venous thromboembolism prophylaxis (VTEP) was advised temporarily. This was followed by B/L sacroplasty after four months, which resulted in significant pain relief and increased mobilisation.

This case showcases the challenges faced in diagnosing fragility fractures in the elderly, diagnostic limitations of DEXA and the potential new role of REMS in the diagnosis of osteoporosis. REMS, being radiation-free, portable and sensitive, may play a role as an alternative or a complementary modality in the diagnosis of osteoporosis and fragility fractures, leading to an improvement of outcomes in vulnerable populations.

## Introduction

Osteoporosis is a pervasive skeletal disorder characterised by decreased bone mineral density (BMD) and the deterioration of bone microarchitecture, which significantly increases fracture risk [1]. The global burden of osteoporosis is substantial, particularly given that osteoporosis is a significant risk factor for fragility fractures. Approximately one in two adult women and one in five men will sustain one or more fragility fractures in their lifetime [2]. In the United Kingdom, approximately 549,000 fragility fractures occur each year, costing the NHS around £4.7 billion a year (around 2.4% of the NHS budget) [3].

According to the World Health Organization (WHO) and NICE CKS guidelines, osteoporosis is diagnosed when BMD falls 2.5 standard deviations or more below the average value for young healthy women, known as a T-score of less than or equal to -2.5 [4]. However, BMD measurement does not assess the structural deterioration in the bone, and consequently, many osteoporotic fractures occur in people who do not have osteoporosis as defined by a T-score of equal to or less than -2.5 [5].

BMD is crucial in determining fracture risk, with lower BMD correlating with a higher risk of fractures [1,5]. The primary diagnostic tool for osteoporosis is dual-energy X-ray absorptiometry (DEXA), which measures BMD and is considered the gold standard for osteoporosis assessment. An emerging alternative is radiofrequency echographic multi-spectrometry (REMS), a noninvasive method that has shown promise in detecting osteoporosis. REMS compares favourably to DEXA, offering sensitivity and specificity of more than 90% in diagnosing osteoporosis [6,7].

Sacral insufficiency fractures (SIFs) are stress fractures occurring in the sacrum. They are particularly relevant in the context of osteoporosis. SIFs often arise in individuals with low BMD and can occur with minimal or no trauma, making them a common complication of osteoporosis [8-10].

Diagnosing SIFs presents significant challenges due to their nonspecific clinical symptoms and the difficulty in detecting them on conventional imaging. Studies indicate that SIFs are frequently missed, with diagnostic rates varying widely [8,10,11]. The complexity of SIF diagnosis highlights the need for heightened clinical awareness and potentially advanced imaging techniques to improve detection rates.

In this case report, we will explore the relationship between osteoporosis and sacral insufficiency fractures, examining the challenges in the diagnosis of osteoporosis and fragility fractures in the elderly, and the role of advanced new imaging modalities such as REMS in assessing BMD and aiding osteoporosis detection.

## Case presentation

A 75-year-old woman presented to the accident and emergency (A&E) with low back pain (LBP) and bilateral (B/L) hip and groin pain on weight bearing. Her past medical/surgical history included osteoarthritis of the B/L hip and right shoulder, B/L total hip replacements and right total shoulder replacement, hypothyroidism, carpal tunnel syndrome, Dupuytren's contracture and hypertension. She had no major risk factors for osteoporosis apart from her age and her postmenopausal status.

She initially had LBP and left hip pain for which she started over-the-counter painkillers, which temporarily helped with the pain. She then started to develop right hip pain on weight bearing. Pain in the LBP and B/L hips was dull in character, non-radiating and mild to moderate in severity and increased on weight bearing. No history of significant trauma was given.

When pain started in both hips and was getting worse in severity, she decided to visit A&E for medical assessment and advice. She had an X-ray of the pelvis and left hip, which showed bilateral total hip replacements in situ with no adverse features, no fractures or dislocations and minor degenerative changes at both sacroiliac joints and marked spondylotic changes at the lower lumbar spine.

Consequently, a diagnosis of lumbar spondylosis was made, and the patient was discharged with oral painkillers such as co-codamol, ibuprofen and eventually gabapentin. She was advised that she is free to mobilise as able and was doing so. No advice of bed rest was given at the time.

On examination, her pain was seven out of 10 (was 5/10 initially when the pain started); the range of movement of both hips was normal and did not induce pain. She had mild to moderate tenderness on palpation of both groins, more on the right side. Her gait was normal, and she was able to perform a straight leg raise B/L with mild discomfort. She was also neurovascularly intact bilaterally, there were no neurological deficits and power was 5/5 in all myotomes. She was able to weight-bear on both legs, her baseline mobility being able to mobilise independently without any aids.

She had an acute blood test done in A&E, which was insignificant. Her inflammatory markers (white blood cell count and C-reactive protein) were within normal range. The doctor did not think of doing osteoporosis screening bloods (vitamin D, calcium, phosphate and parathyroid hormone levels).

**Figure 1:**
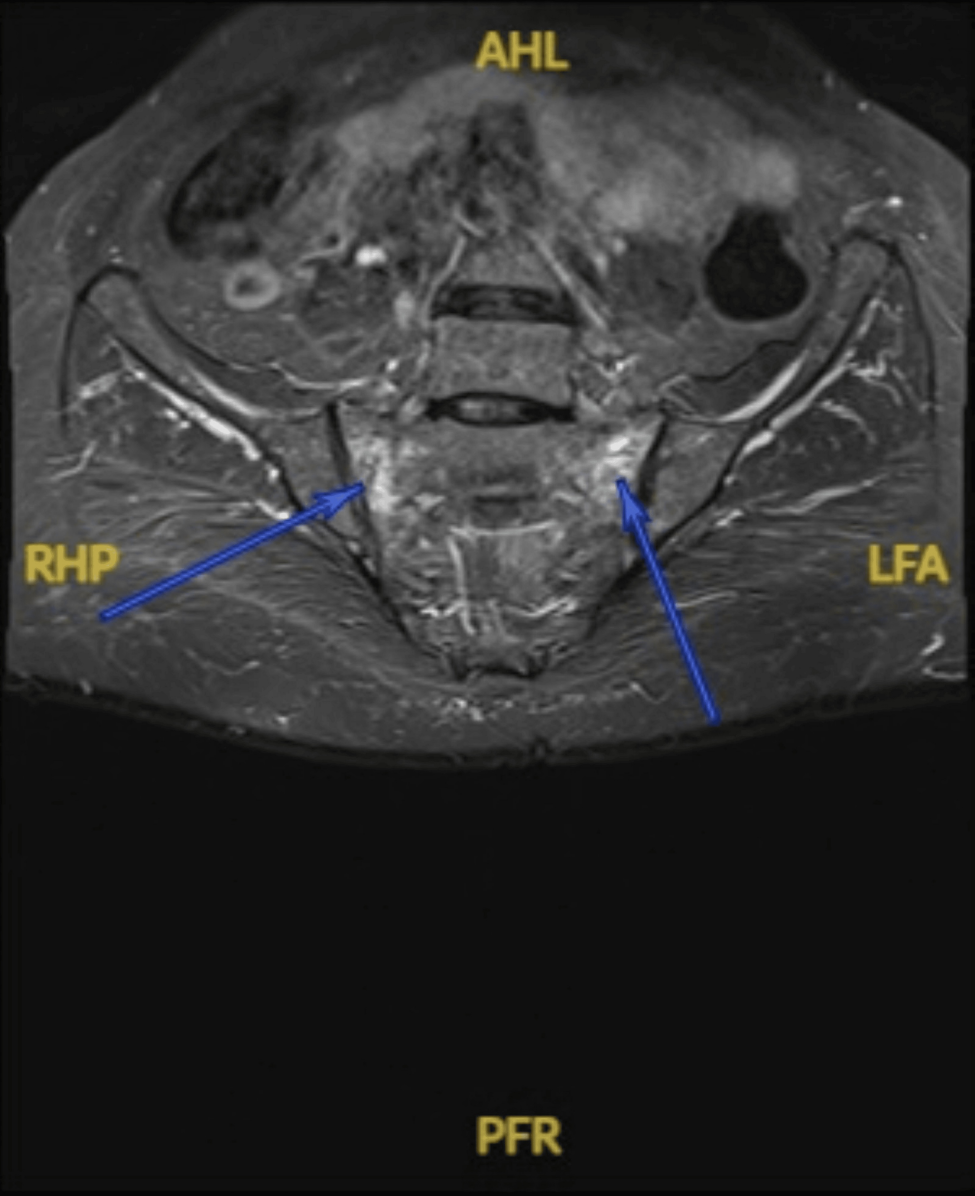
MRI of the sacrum, T2-weighted coronal images, showing bilateral sacral ala insufficiency fracture mostly in zone 1 between S1 and S3. AHL, anterior high left; RHP, right high posterior; LFA, left fossa anterior; PFR, posterior fossa right

Three months had passed from her A&E visit, and her pain did not get any better. Concerned about the pain and frustrated with her management plan, she opted to have a private check-up where she had an MRI of the sacrum done, which showed bilateral insufficiency sacral ala fractures (Figure 1). The MRI was reported as 'bilateral insufficiency type sacral ala fractures. These are mainly limited to zone 1 between S1 and S3, with probable extension into zone 2 at the right S2 level. No significant displacement'.

A DEXA scan of her lumbar spine was done a month later, as there was suspicion of osteoporosis. Surprisingly, the T-score of our 75-year-old patient was -0.9, the bone mineral density (BMD) was 0.949 g/cm^2 ^and the Z-score was 1.4.These DEXA scan results suggest normal bone density and make osteoporosis seem unlikely.

Two noteworthy points were mentioned in her DEXA scan report.

Firstly, femoral neck DEXA could not be performed due to the patient having bilateral total hip replacements done. Hence, DEXA scan follow-up was not advised 'due to the lack of anatomical sites to accurately measure the BMD', as per the report.

Secondly, it was mentioned that spine DEXA scans become less reliable after the age of 70 years due to the degenerative process.

As the patient had bilateral sacral ala fracture with no history of trauma or falls, there was a strong suspicion of osteoporosis, despite a DEXA scan suggesting normal BMD. Hence, she underwent a REMS ultrasound scan, which showed a fragility score of 76 (range: 0-100 {maximum fragility of bone structure}), suggesting osteoporosis [6]. She was then started on bone-protective medication of combined colecalciferol 400 IU/calcium carbonate 500 mg chewable tablets, one tablet taken twice daily every day for life.

**Figure 2:**
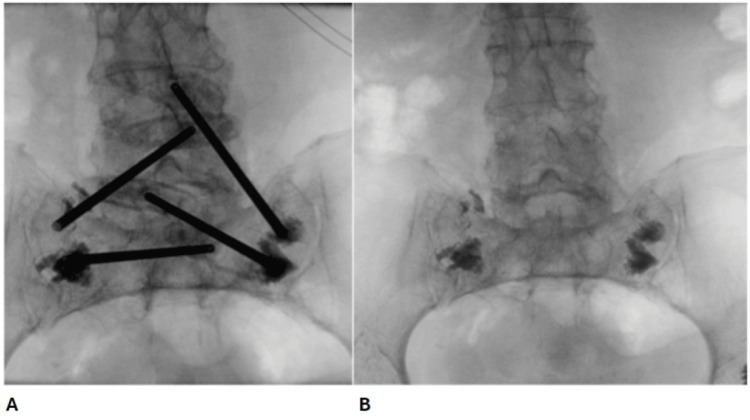
Fluoroscopy images taken during the patient's bilateral sacroplasty surgery. (A) Fluoroscopy image showing bone needles injecting bone cement at the site of fractures during the surgery. (B) Fluoroscopy image showing bone cement injected in the bilateral sacral ala at the site of fractures at the end of the surgery.

Following the MRI, the patient was advised to have bed rest with painkillers and VTE prophylaxis prescribed. She eventually had bilateral sacroplasty done at a major trauma centre (Figure 2A, 2B), around four months after the MRI of the sacrum showing fractures.

At her one-month follow-up in the outpatient clinic, she mentioned that her pain had almost completely resolved. Her mobility returned to baseline after four weeks of physiotherapy following the surgery.

## Discussion

This case highlights two important learning points: the difficulty and importance of timely diagnosing and treating sacral insufficiency fractures (SIFs) in the elderly population and how new and exciting technology, such as REMS, can be used as a complementary diagnostic tool in measuring BMD.

The sacrum serves as the connection point between the spine and the pelvis, bearing the weight of the upper body and providing essential stability to the pelvic ring during weight bearing. It is closely associated with vital structures, including nerves, blood vessels and pelvic organs. A sacral fracture can significantly impact mobility and may be accompanied by various related injuries.

Non-traumatic SIFs secondary to osteoporosis are one of the most missed and ignored fractures in the elderly population [8,11]. This can lead to increased mortality according to some literature [9] (however, causes of increased mortality in patients with SIFs can be multifactorial, as most patients are elderly and have other issues and comorbidities [12]). 

Considering the anatomical significance of os sacrum [8], the increased mortality associated with SIFs and complications associated with the mainstay conservative treatment resulting in decreased mobility [9], it is important that these fractures are not taken lightly and diagnosed and managed properly and timely. One of the factors that helps in raising clinical suspicion and diagnosing SIFs is a diagnosis of osteoporosis, for which purpose a DEXA scan failed us in this case.

A learning point we can appreciate from this case is to investigate further into complaints of pain, especially in the elderly. When the X-ray showed no fractures, it would have been justified for the A&E team to order further scans, such as an MRI of the pelvis, to confidently rule out any fractures or any other pathologies. Perhaps the patient's lack of severity of B/L hip pain, her ability to weight-bear and the lack of osteoporosis as a comorbidity did not raise clinical suspicion and concern for the A&E team to investigate further.

A timely diagnosis of osteoporosis can also help prevent fragility fractures by commencing osteoporosis treatment. A diagnosis of osteoporosis will also help us clinically suspect insufficiency fractures, which can easily be missed on initial scans in the elderly population. Appreciating the importance of osteoporosis diagnosis, should we rule out osteoporosis with a normal T-score on DEXA despite clinical suspicion? Some clinicians might be tempted to do so, resulting in potential patient harm.

A new and exciting tool to help us analyse BMD and aid in diagnosing osteoporosis is radiofrequency echographic multi-spectrometry (REMS). It is a radiation-free, portable, quick and easily repeatable technology that assesses the bone structure and fragility fracture risk.

**Figure 3:**
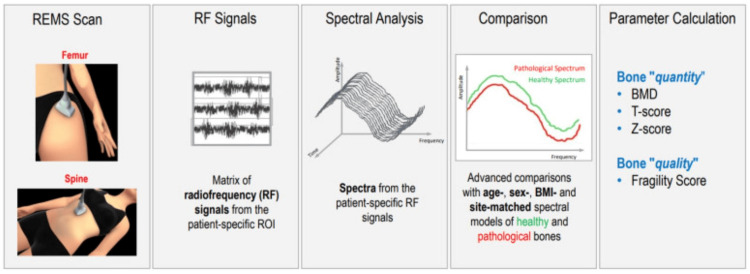
An illustration of radiofrequency echographic multi-spectrometry (REMS) work. Copyright/license: this figure has been adopted from 'Radiofrequency echographic multi spectrometry (R.E.M.S.): new frontiers for ultrasound use in the assessment of bone status-a current picture', by Al Refaie A, Baldassini L, Mondillo C, et al., 2023, Diagnostics (Basel), 13(10), 1666 [13], which is an open-access article distributed under the terms and conditions of a Creative Commons CC By license (https://creativecommons.org/licenses/by/4.0/). No changes were made. ROI, region of interest; BMD, bone mineral density

REMS relies on ultrasound waves, together with advanced spectral analysis. The REMS scan is carried out with portable devices, usually targeting the lumbar spine and femoral neck. The clinician places the ultrasound probe, targeting the lumbar spine or femoral neck and regulating the depth and the focus of the transducer to visualise the interface of the target bone. The receiver then captures and measures the quality of reflected ultrasound waves (radiofrequency signals) from the bone, which then undergoes spectral analysis by the software. The data is then compared to reference population data (from osteoporotic and healthy populations), and quantitative parameters (such as BMD, T-score and Z-score) and qualitative parameters (fragility score) are calculated [6,13]. This is illustrated in Figure 3.

There is growing literature supporting the use and benefits of using REMS as compared to DEXA scans. However, REMS is not currently recommended in any of the guidelines.

One of the biggest advantages of REMS over DEXA would be that it is essentially an ultrasound scan. This means that it is easily repeated, portable and radiation-free. In contrast, DEXA scans are an ionising scan, meaning that it exposes patients to radiation, and because of this, they are not easily repeatable. Also, DEXA scans are large scanners, which are not portable; patients would need to be transferred to the scanners, which can raise logistical issues.

The accuracy of REMS in diagnosing osteoporosis was well defined in an Italian study done by Di Paola et al. on women aged 51-70. It showed a sensitivity of 91.5% at the femoral neck and 91.7% at the lumbar spine and specificities of 91.8% at the femoral neck and 92.0% at the lumbar spine [7]. This shows us that the accuracy of REMS is comparable to that of DEXA scans and can be relied on. Perhaps more studies comparing the accuracy and precision of both scans would give us a clearer idea.

Another advantage of REMS over DEXA is the automatic consideration of artefacts from metal structures, calcifications, osteophytes, vertebral fractures and other artefacts. As recent studies have demonstrated, this may lead to more precise measures of BMD [6,14-16]. Due to these shortcomings of DEXA scans, quantitative MRI and CT scans are used to assess for osteoporosis, especially in patients with degenerative spines [17,18]. REMS potentially could be utilised instead of DEXA scans in these cases, to provide better or the same assessment of osteoporosis without the need for radiation exposure to patients or logistics issues (although it may not provide the same quality of images as MRI/CT).

REMS is increasingly in use because of its safety and repeatability qualities in populations in whom ionising scans, such as DEXA, would be risky to use. These characteristics are showcased in the study by Forcignanò et al.: 'Short-term monitoring of denosumab effect in breast cancer patients receiving aromatase inhibitors using radiofrequency echographic multi-spectrometry (REMS) technology on femoral neck' [19]. The study was done to show the effect of denosumab on BMD in patients with breast cancer taking aromatase inhibitor therapy (known to cause bone loss and increase the risk of osteoporosis). REMS was used as an alternative to DEXA, as scans were repeated every six months. The author mentions the following: 'This study showed the feasibility of short-term follow-up using REMS lumbar spine scans at 6-month time steps' [19].

Another case where REMS was preferred due to its safe nature is by Lombardi et al., titled 'A case report of post-pregnancy osteoporosis monitoring by means of REMS technology' [20]. In this case study, a 33-year-old woman was diagnosed with the rare pregnancy-associated osteoporosis (PAO) using a REMS scan. It is argued that PAO can be missed as most clinicians will be sceptic to using ionising scans such as DEXA during pregnancy and attributing back pain to pregnancy (similarly to our case where back pain was attributed to age-related spinal degenerative disease). This case study suggests to us that REMS can be a safe and efficient alternative tool to use and highlights the need for reviewing our osteoporosis-diagnosing practice [20].

## Conclusions

In our case, our patient was discharged with oral analgesics, free to fully weight-bear, no follow-up, no osteoporosis treatment and no proper explanation for pain. We predict that a lot of patients with undiagnosed SIFs would have gone through similar experiences, although we have no statistics or literature to support this. One of our aims in this case report is to highlight the challenges of diagnosing insufficiency fractures, such as SIFs, in elderly patients so that our growing elderly patients can be provided with better care instead of repeated oral analgesics to mask the problem.

What stands out in this case is the fact that the patient had bilateral SIFs, yet the MRI of the pelvis was delayed, and the DEXA scan showed normal bone mineral density. The fact that the patient required B/L sacroplasty and made a full recovery postoperatively again signifies the importance of being careful not to miss SIFs in the elderly population. This case report also underscores the importance of integrating new technologies such as REMS into clinical practice to improve diagnostic accuracy and patient outcomes. By adopting such tools, healthcare providers can better diagnose and manage osteoporosis and related fragility fractures, ultimately leading to more effective treatment and a reduction in complications associated with missed diagnoses. This is especially relevant in the elderly population and patients with hip prostheses where conventional scans such as DEXA may not be as reliable.
